# Outbreak of autochthonous West Nile virus infection in Lazio region, Italy, July to August 2025: preliminary investigation

**DOI:** 10.2807/1560-7917.ES.2025.30.35.2500634

**Published:** 2025-09-04

**Authors:** Ilaria Mussetto, Andrea Bongiovanni, Francesca Colavita, Cristina Giambi, Marcello Giovanni Sala, Cosmo Del Borgo, Fabrizio Carletti, Maria Teresa Scicluna, Alessandro Zerbetto, Angela Corpolongo, Federico Romiti, Maria Beatrice Valli, Stefania Vaglio, Roberto Giammattei, Paola Scognamiglio, Gabriella De Carli, Alessandro Agresta, Claudio De Liberato, Giuseppe Di Luzio, Florindo Micarelli, Emanuele Nicastri, Andrea Siddu, Valeria Ficarelli, Enrico Girardi, Fabrizio Maggi, Francesco Vairo, the Lazio WNV Outbreak Group, The Lazio WNV outbreak group, Martina Rueca, Claudia Minosse, Cesare Ernesto Maria Gruber, Gabriella Rozera, Silvia Meschi, Silvia Cammisa, Licia Bordi, Roberta Sciamanna, Silvia Biancone, Valeria Ferraioli, Loredana Aleo, Sabrina Coen, Andrea Mariano, Giovanna De Angelis, Maria Lorena Rossi, Dario Turco, Francesco Censi, Valentina Vantaggio, Giorgio Nicolò Malatesta, Maria Concetta Fusco, Edoardo Carnevale, Gilda Tonziello, Raffaella Marocco, Tiziana Tieghi, Adele Magliano, Lucy Nicole Papa Caminiti, Roberto Nardini, Antonella Cersini, Ida Ricci, Francesca Rosone, Andrea Carvelli

**Affiliations:** 1 National Institute for Infectious Diseases “Lazzaro Spallanzani” IRCCS, Rome, Italy; 2 Department of Prevention, Local Health Authority Latina, Latina, Italy; 3 Istituto Zooprofilattico Sperimentale del Lazio e della Toscana “M. Aleandri”, Rome, Italy; 4 Infectious Diseases Unit, Santa Maria (SM) Goretti Hospital, Sapienza University of Rome, Latina, Italy; 5 Lazio Regional Blood Center, Italy; and Department of Molecular Medicine, Sapienza University, Rome, Italy; 6 Department of Prevention, Local Health Authority Roma 6, Ariccia (Rome), Italy; 7 Directorate for Health and Social Policy, Lazio Region, Rome, Italy; 8 Department of Prevention, Local Health Authority Frosinone, Frosinone, Italy; 9 The members of the group are listed under Collaborators

**Keywords:** West Nile virus, Italy, Outbreak, Autochthonous, Arbovirosis, One Health

## Abstract

In July–18 August 2025, 171 autochthonous cases with West Nile virus (WNV) infection were confirmed in Lazio, Italy: four asymptomatic blood donors, 110 with WNV fever, 57 with neuroinvasive syndrome and nine deaths. WNV lineage 2 was detected in two neuroinvasive cases. Infection with WNV was confirmed in 28 horses, five crows and a *Culex pipiens* pool. We present the preliminary epidemiological and phylogenetic analysis of the outbreak and the public health measures taken within a One Health approach.

Starting in mid-July 2025, an autochthonous outbreak of West Nile virus (WNV) infection was detected in the Lazio region, Italy, where human cases had not previously been reported. We describe the epidemiological and laboratory investigations following the detection of the first cases, as well as the public health response to this outbreak.

## Event description

On 14 July 2025, the Regional Reference Laboratory (RRL) at the Lazzaro Spallanzani National Institute for Infectious Diseases, detected WNV IgM antibodies in two patients with meningoencephalitis and hospitalised in the infectious diseases department of the referral hospital in the province of Latina. Both patients were from the province of Latina and had no reported travel or other known foreign exposure in the 21 days before symptom onset. Therefore, they were categorised as autochthonous cases. On 15 July, following molecular investigations and further serological analysis of cerebrospinal fluid (CSF) samples, both cases were confirmed based on the European Union (EU) case definitions [1].

On 16 July, the Istituto Zooprofilattico Sperimentale del Lazio e della Toscana (IZSLT) identified WNV IgM antibodies in a sample from a horse with neurological symptoms and living in the province of Latina, and WNV positive real-time (RT) PCR results in a pool of mosquitoes collected on 9 July in the municipality of Pontinia, in the province of Latina.

Following the initial confirmation of these cases, the regional public health authorities coordinated the implementation of public health interventions, fully consistent with the national [2] and regional [3] plan for arboviral infections and reinforced by extraordinary measures. Surveillance activities were promptly intensified in all regional hospitals and among general practitioners, recommending the inclusion of WNV infection in the differential diagnosis of persons with fever and/or neurological symptoms. Retrospective case reviews and syndromic monitoring in emergency departments, focusing on neuroinvasive symptoms, were conducted. Surveillance was coordinated within a One Health framework, strengthening active and passive surveillance of local equine and bird species across the region.

## Epidemiological investigation

**Figure 1:**
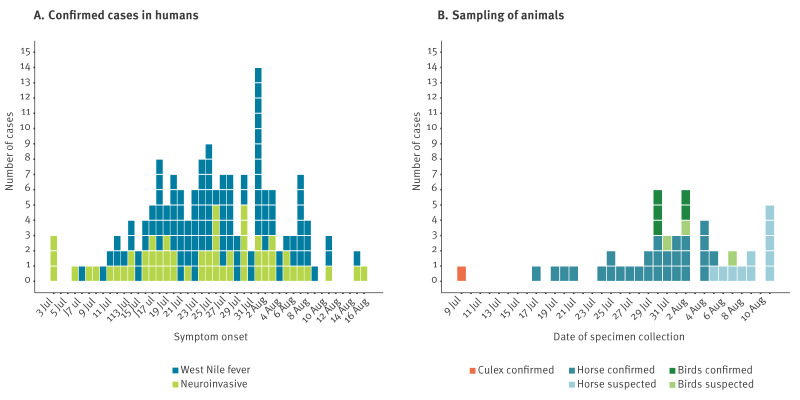
Timeline of symptom onset of human cases with West Nile virus infection (n = 167) (A) and sampling of animals (B), Lazio region, Italy, July–18 August 2025

By 18 August 2025, WNV infection was confirmed in 171 patients with exposure in Lazio (167 notified by Lazio and 4 by other regions). Of these, 110 (64.3%) had WNV fever syndrome (WNF), 57 (33.3%) a neuroinvasive syndrome (WNND) and 4 (2.3%) were asymptomatic blood donors. Figure 1A shows the epidemic curve of the 167 symptomatic cases. The median age of the symptomatic cases was 62 years (interquartile range (IQR): 46.5–73 years) (Table). Cases with WNND were older than those with WNF (76 vs 53 years; p < 0.001). Almost all WNND cases (56/57) were hospitalised, and 10 patients were treated in the intensive care unit (ICU). Most WNF cases (n = 74; 67.3%) did not need hospital treatment. Comorbidities (22/57 vs 23/110 patients; p = 0.023) were more common and case-fatality rates (8/57 vs 1/110 patients; p = 0.001) were higher in cases with WNND than in those with WNF. The patient with WNF, who died, had a history of organ transplantation.

**Table:** Demographic and clinical characteristics of confirmed symptomatic human cases of West Nile virus infection, Lazio region, Italy, July–18 August 2025 (n = 167)

Characteristics	Clinical manifestation						
	Neuroinvasive disease (n = 57)		WNV fever (n = 110)		p value^a^	Total	
	n	%	n	%		n	%
Age (years)							
Median	76		53		< 0.001	62	
IQR	68–83		40.2–65.8			46.5–73	
Sex							
Female	22	38.6	54	49.1	0.259	76	45.5
Male	35	61.4	56	50.9		91	54.5
Comorbidities							
Yes	22	38.6	23	20.9	0.023	45	26.9
Hospitalisation							
General ward	46	80.7	36	32.7	< 0.001	82	49.1
Intensive care unit (ICU)	10	17.5	0	0		10	6.0
Not hospitalised	1	1.8	74	67.3		75	44.9
Outcome							
Deceased	8	14.0	1	0.9	0.001	9	5.4

IQR: Interquartile range; WNV: West Nile virus.

^a^ Pearson’s chi-square test or Fisher’s exact test for categorical variables and Wilcoxon rank-sum for continuous variables.

Most cases (n = 155) were considered having been exposed in municipalities in the province of Latina, mainly in the city of Latina and in smaller coastal or rural towns. Since 24 July, cases were also notified in the province of Rome, involving municipalities along the coast, including Anzio and Nettuno near the border with Latina. Cases were subsequently identified further inland, and, in the following days, in municipalities of the province of Frosinone as well as in a coastal district on the outskirts of Rome.

**Figure 2:**
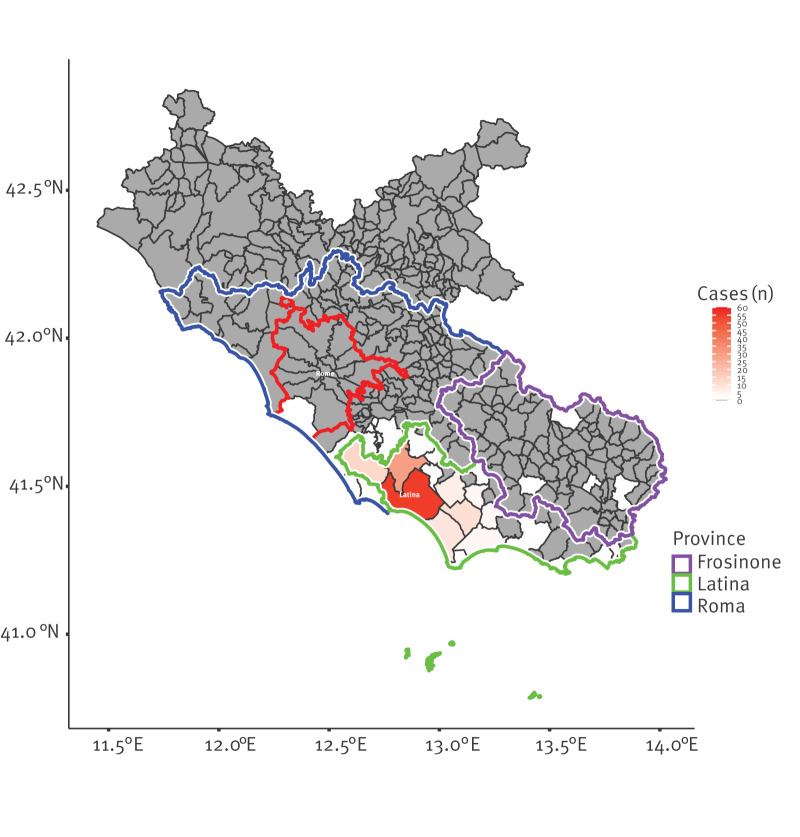
Probable places of exposure of confirmed symptomatic human cases of West Nile virus infection, Lazio region, Italy, July–18 August 2025 (n = 167)

Based on the case interviews, probable exposure occurred in extra-urban areas for 106 (62%) cases, at the border between urban and suburban areas for 22 (12.9%), and in urban areas for 43 (25.1%) cases. The spatial distribution of cases is shown in Figure 2.

The figure presents the map of Lazio, Italy, divided by municipalities (the city of Rome is outlined in red, divided into districts). The coloured boundary lines indicate the provincial borders of Rome (blue), Latina (green) and Frosinone (purple).

Municipalities with no notified cases are marked with grey colour. Municipalities with notified cases range from white (one case) to deep red (60 cases). Latina municipality had the highest number of cases (55 cases), followed by Cisterna di Latina (30 cases), and additional cases in the surrounding municipalities.

As part of the animal surveillance, WNV infection was confirmed in several municipalities, mainly in the provinces of Latina and Rome. By 18 August, the infection was confirmed in 28 horses: 25 were unvaccinated and tested positive with IgM ELISA, and three were RT-PCR positive. The virus was also confirmed with RT-PCR in five crows collected via thinning activities. One *Culex pipiens* mosquito pool of 74 tested was RT-PCR positive (Figure 1B). By 2 September, WNV infection was confirmed in an additional 11 horses and three crows.

## Laboratory investigation

Between 14 July and 18 August, 976 samples from 464 patients were analysed with molecular assays RealStar WNV RT-PCR (Altona Diagnostics GmbH, Hamburg, Germany) or cobas WNV test (Roche, Basel, Switzerland), followed by an in-house pan-flavivirus nested RT-PCR targeting the NS5 gene for sequencing. Of these 976 samples, 132 of 425 (31%) plasma samples were positive, 5 of 24 serum samples, 166 of 396 (42%) urine samples and 7 of 134 (5%) CSF samples.

A total of 397 samples (343 serum, 54 CSF) were tested for anti-WNV IgM/IgG antibodies using either West Nile Virus VirClia monotest (VirCell Microbiologists, Granada, Spain) or indirect immunofluorescence assays Arbovirus Profile 3 (Euroimmun, Lübeck, Germany).

**Figure 3:**
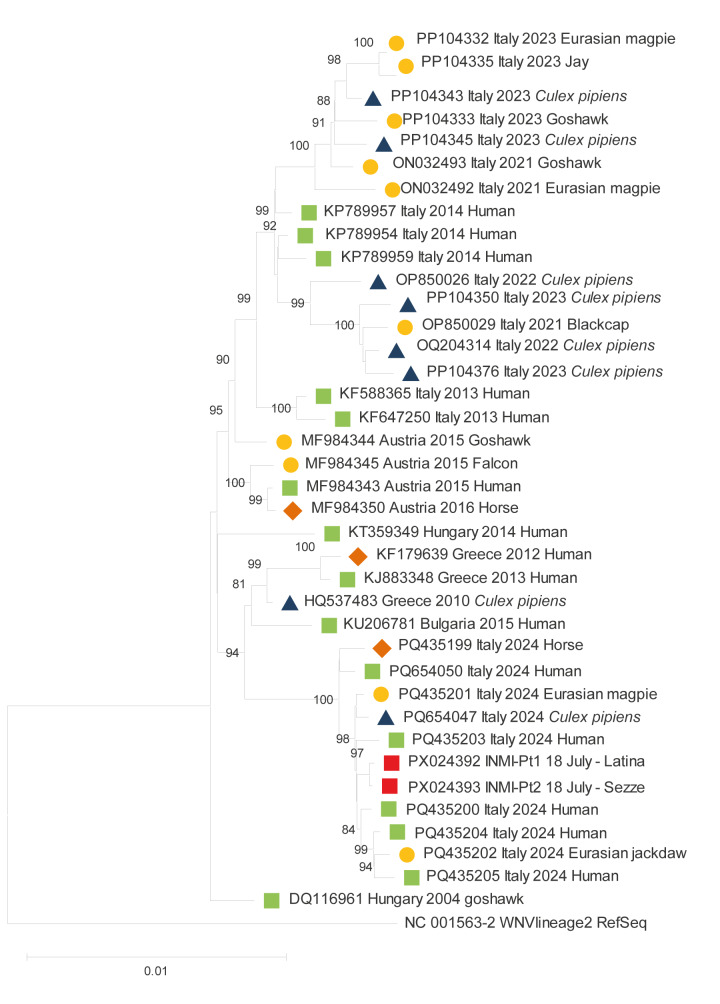
Phylogenetic analysis of full-length genome sequences from two autochthonous cases of West Nile virus infection in Lazio, July 2025 and sequences from humans (n = 15), birds (n = 11) and horses (n = 2)

Samples negative for WNV RNA but positive for anti-WNV antibodies were referred for confirmatory neutralisation testing in BSL-3 laboratories of the RRL of the Lazzaro Spallanzani National Institute for Infectious Diseases. Differential diagnosis was also performed by testing other related orthoflaviviruses (i.e. Usutu virus; dengue virus). Full-length WNV genome sequencing was performed on urine samples from two patients using a Next Generation Sequencing (NGS) amplicon-based approach on the Ion Gene Studio S5 Prime system (Thermo Fisher Scientific, Waltham, the United States) with 99.3% genome coverage. The analysis of polyprotein confirmed the identity of 98.5% with lineage 2 strains clustering with sequences reported in 2024 in central-southern Italy (Campania region) (Figure 3).

WNV: West Nile virus.

Sequences presenting the two autochthonous cases described in this rapid communication are marked with a red square, other human cases of WNV infection belonging to the phylogenetic cluster are marked with green square, sequences from mosquitoes with blue triangle, sequences from birds with yellow circle and sequences from horses with orange rhombus. Bootstrap values > 90 are reported aside the corresponding nodes.

## Public health measures

Vector control measures targeted *Cx. pipiens*, the main WNV vector in Italy, using larvicides, adulticides and source reduction. Treatments were carried out within 200 m of the place of the probable exposure of each human case: larvicides in rural environments, and both larvicides and adulticides in urban areas. When there was a cluster of more than three cases in an urban area, the treatments were extended to a larger area including all cases of the cluster, by building polygons around the more external ones.

Public risk communication was a key factor for increasing awareness of personal protective behaviour and community participation in mosquito control; in the province of Latina, information material was disseminated through the national platform (AppIO) [4], reaching people with this app installed (29% of the local population). Healthcare professionals received specific training to improve clinical suspicion, diagnostic accuracy and prompt case reporting.

Safety protocols for organ, tissue, haematopoietic stem cell and blood donation [5] were strengthened. On 16 July, nucleic acid testing (NAT) for WNV was implemented for donors who had stayed at least one night in Latina province within the previous 28 days [6], and on 24 July, screening was expanded to all blood donations in the Lazio region [7].

Furthermore, entomological monitoring, serological surveillance of sentinel horses, and surveillance of synanthropic bird species were strictly aligned with the regional surveillance plan already in place. These activities were recalibrated and intensified to enable early detection of viral circulation and prompt activation of control measures. In particular, local public health authorities were urged to enhance synanthropic bird thinning activities along with passive surveillance to monitor the potential onset of a local WNV transmission cycle, with special attention in areas with no cases detected in humans or animals. Once WNV infection had been confirmed in resident synanthropic birds or in horses in areas not previously affected, alert levels for the identification of probable human cases were raised, both in emergency departments and among general practitioners. This was supported by rapid information initiatives, including webinars targeted at healthcare professionals, to ensure timely recognition, reporting, and response to potential infections.

## Discussion

Since 2008, WNV infection has been annually detected in northern Italy, especially in the Po river valley [8], while Lazio has not previously notified autochthonous human cases. Between 2009 and 2024, WNV infection was sporadically confirmed in 21 horses in the provinces of Viterbo, Latina and Rome, the latest detected in 2020, without evidence of continuous circulation of the virus [9], suggesting its seasonal introduction from endemic regions via migratory birds.

By 27 August 2025, Italy had 430 confirmed WNV human cases, including 193 (44.9%) neuroinvasive cases and 27 deaths [10]. The first autochthonous case during the 2025 transmission season was reported in July in Modena (Emilia-Romagna), with further cases occurring in 10 other Italian regions [10].

Indeed, the 2024–25 winter was characterised by unusual mild temperatures, likely triggering an early activation of overwintering *Cx. pipiens* females from diapause. This may have accelerated their reproductive cycle, resulting in a larger vector population by spring, when birds carrying the virus from potentially endemic zones arrived in the area. This convergence, coupled with a shortened extrinsic incubation period due to elevated temperatures, likely facilitated earlier and more intense viral circulation in areas with a high density of local amplifier bird species, ultimately sparking the onset of the epidemic.

The Latina province, where most cases occurred, was already considered at high risk for WNV because of previous findings in animals and an environment particularly rich of larval breeding sites suitable for *Cx. pipiens* development, such as drains, ditches and small swamps. The virus was detected in all components of its biological cycle—birds and mosquitoes—even at the periphery of the epidemic zone, suggesting a sustained transmission in the area, thus highlighting the need for continued attention and enhanced surveillance.

Consistent with recent European epidemiological data [11], the genomic characterisation identified the lineage 2 as the cause of the WNV outbreak we described here, in humans, birds, mosquitoes and equids. The phylogenetic connection with the sequences reported in 2024 in central-southern Italy supports the hypothesis that the virus responsible for the current outbreak in Lazio could be related to the strains circulating the previous year. Additional investigations will be required to better characterise the phylogenetic origin of the WNV lineage implicated.

## Conclusion

The outbreak in Lazio Region is ongoing, with 216 confirmed cases (68 WNND) at the time of writing. Preventive and control measures according to arbovirus response plans, as well as extraordinary measures, are in place using a One Health approach that incorporates human and animal surveillance. The current outbreak in Lazio and in other neighbouring regions, alerts to the expanding circulation of WNV in new areas of Italy. These findings underscore the need for further research to guide future public health measures and assess whether existing surveillance systems require improvement for higher sensitivity. In parallel, during the vector season, it is essential to raise awareness among clinicians to include WNV in the differential diagnosis of febrile and/or neuroinvasive cases.

## Data Availability

Sequence data obtained in this study have been submitted to GenBank under accession numbers: PX024392, PX024393. Further data that support the findings of this study are available from the corresponding author upon reasonable request.
